# Mitigation Strategies for African Swine Fever Virus Biosecurity: From Virus Inactivation to Pig Health

**DOI:** 10.1155/tbed/7162567

**Published:** 2025-06-23

**Authors:** Joshua A. Jackman, Bo Kyeong Yoon, Charles C. Elrod

**Affiliations:** ^1^School of Chemical Engineering and Translational Nanobioscience Research Center, Sungkyunkwan University, Suwon, Republic of Korea; ^2^School of Biomedical Engineering, Chonnam National University, Yeosu, Republic of Korea; ^3^Natural Biologics Inc., Newfield, New York, USA

**Keywords:** African swine fever virus, antiviral, disinfectant, mitigant, thermal inactivation, virucidal

## Abstract

The African swine fever virus (ASFV) is a major global threat affecting pork production and strengthening biosecurity practices is an urgent priority, especially given the paucity of effective vaccines and antiviral drugs. Mitigation strategies focused on virus inactivation play an important role in controlling ASFV and there is growing recognition that multipronged mitigation strategies can not only achieve rapid decontamination of ASFV-exposed materials in different environmental settings but also support pig health by minimizing disease symptoms and preventing lateral transmission. Herein, we critically analyze the latest progress in developing thermal, chemical, and physical strategies to stop ASFV based on heat treatment, chemically reactive disinfectants, and physically disruptive mitigants. Our focus is on introducing ASFV-specific data that supports the use of different mitigation strategies in particular contexts and analyzing the corresponding inactivation mechanisms behind each strategy. In closing, we also discuss emerging innovation possibilities in the ASFV mitigation testing space and provide a forward-looking viewpoint of outstanding scientific questions and future research needs.

## 1. Introduction

The African swine fever virus (ASFV) is responsible for a highly lethal hemorrhagic disease that affects domestic and wild pigs worldwide and is a major threat to the pork production industry [1, 2]. This threat is highlighted by the ASF epidemic in China—the world's largest pork producer—that occurred in 2018–2019 and caused an over 40% reduction in the pig population nationwide due to pathogenic infections and preventative culling efforts [3–5]. This rapid decline caused a marked decrease in pork supplies as well as a spike in pork prices globally, which further underscores the risk to food security [6]. Additional ASFV outbreaks have also occurred repeatedly throughout Southeast Asia, Europe, and Central Asia [7–9]. There are also concerns about the risk of ASFV spreading to currently ASF-free regions such as the United States, which would disrupt food supply chains and cause significant economic damage [10]. Given these risks and challenges, the primary goals of ASF biosecurity measures are to prevent transboundary spread to ASF-free regions and to contain outbreaks in endemic regions.

ASFV control and prevention strategies differ significantly between ASF-free and endemic regions due to their distinct risk factors and containment priorities. In ASF-free regions, biosecurity efforts mainly focus on strict import controls, border surveillance, and early detection to prevent virus introduction [11–14]. Preventative measures include decontamination protocols on farms and restrictions on high-risk imports such as feed ingredients and pork products from ASF-endemic regions [15]. In contrast, ASF-endemic regions require a multi-tiered containment strategy that integrates biosecurity with active and passive surveillance, rapid diagnostics, and outbreak response measures [16–18]. Key priorities include strict farm biosecurity, routine monitoring of pig herds and wild boar populations, rapid culling of infected animals, and movement restrictions to limit ASFV spread [19, 20]. In addition, secondary spread within farms, which is caused by infected pigs transmitting the virus to healthy animals, is a major challenge.

The need for effective biosecurity practices is intensified by limited vaccine options to prevent infection and the lack of approved antiviral drugs [21, 22]. As a result, current biosecurity efforts mainly focus on inactivating virus contaminants on potential transmission vectors in order to prevent virus spread to healthy pigs [23, 24]. These vectors include feed and feed ingredients, drinking water, animal carcasses, surfaces, and fomites such as equipment, vehicles, and the clothing and footwear of farm and delivery workers, many of which have been implicated in causing ASFV spread [25–31]. The relative importance of these vectors depends on whether a region is ASF-free or endemic. For example, infectious ASFV can persist in feed, feed ingredients, and pork products for extended periods, which makes them significant risks for transboundary spread into ASF-free regions [32]. In those cases, the major focus is on preventing ASFV entry into farms. On the other hand, in endemic regions, transmission from ASFV-infected wild boars and their carcasses to healthy pigs is a critical risk [33] and the major focus is on curtailing ASFV spread to limit outbreaks. In addition, on farms in endemic regions, ASFV can be transmitted from infected pigs to healthy pigs by direct contact or indirect routes such as feces and bodily secretions as well as by contaminated swill or drinking water [34].

Given the wide range of transmissibility risks in ASF-free and endemic regions, a differentiated mitigation strategy is needed to effectively inactivate virus contaminants in diverse environmental settings ranging from soils and material surfaces to complex biological matrices such as feed and even potentially inside animals. Within this scope, an emerging view of pathogen mitigation is shifting from immediate decontamination of potential transmission vectors to a more holistic view of animal health whereby decontamination is one component alongside other needs such as enhancing immune health to ward off disease and preventing lateral transmission [35]. This viewpoint highlights how ASFV mitigation strategies fit within broader biosecurity frameworks, complementing other elements such as strict quarantine protocols, herd management, controlled animal movement, and regional coordination of disease monitoring. These strategies can support synergistic approaches to achieve ASF disease prevention and control.

The objective of this review is to introduce the latest mitigation strategies aimed at controlling ASFV based on various inactivation strategies that are divided into the following categories: (1) thermal treatments; (2) chemically reactive disinfectants; (3) physically disruptive mitigants. We begin by introducing the current status of ASFV vaccine and antiviral drug development in order to discuss capability gaps that mitigation strategies can help address and then introduce the unique structural aspects of ASFV that make it challenging to stop compared to other livestock viruses. Detailed coverage of different ASFV mitigation strategies is then provided and we pay particular attention to the application context and corresponding inactivation mechanisms associated with each strategy. Together, these efforts lead to building a holistic view of how different mitigation strategies fit together to facilitate a comprehensive biosecurity framework, and we also discuss ongoing advances in developing ASFV surrogate models to expand mitigation testing possibilities.

## 2. Current Status of ASFV Countermeasures

**Figure 1:**
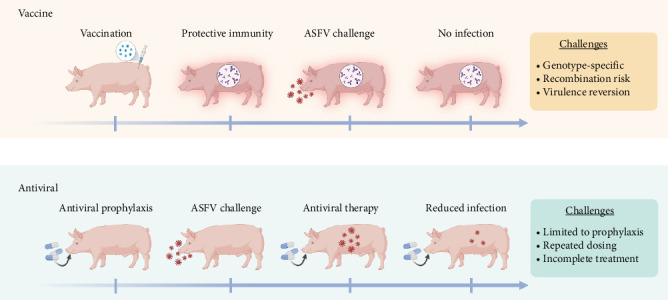
Overview of ASFV vaccine and antiviral capabilities. (A) Vaccines are focused on preventing infection from virulent ASFV strains but face challenges such as limited protection against different ASFV genotypes, potential recombination with circulating strains to create new mutant strains, and risk of nonvirulent vaccine strains mutating and becoming virulent. (B) Antiviral strategies are focused on delivering medicines to infected pigs in order to inhibit ASFV replication and reduce clinical symptoms. While some antiviral drugs can work against different ASFV genotypes, this approach faces challenges such as limited success in prophylactic settings (drug dosing starts before infection occurs) and has not been demonstrated in therapeutic settings (infection occurs before drug dosing starts), the antiviral drug needs to be administered to the infected pig repeatedly, and the treatment outcomes are often incomplete in terms of disease reduction. Created in BioRender. Jackman, J. (2025) https://BioRender.com/3kllv4e. ASFV, African swine fever virus.

An ideal strategy to prevent ASFV infections would be widespread vaccination of pigs worldwide. However, ASFV vaccine options are limited and largely still in the development stage. Likewise, antiviral drugs to thwart ASFV infections prophylactically are being explored at the research stage but are not approved or commercially available. Considering these issues, there is a high priority placed on developing effective mitigation strategies to blunt ASFV spread based on disinfection methods. To illustrate this need, Figure 1 presents the current status of ASFV vaccine and antiviral countermeasures under development, and the listed challenges highlight opportunities where different mitigation strategies can be employed to fill in capability gaps.

### 2.1. Vaccines

The development of an effective ASFV vaccine to prevent viral infections is a major goal of ASFV research (Figure 1A). The basic steps involve vaccinating healthy pigs with ASFV antigens and helping them to build up protective immunity to ward off future exposure to more virulent ASFV strains and prevent reinfection. Various ASFV vaccine platforms have been explored, including inactivated (killed), recombinant protein-based subunit, nucleic acid (DNA), vector, and live-attenuated vaccines (LAVs), and each platform has particular advantages and challenges.

Killed vaccines, which consist of inactivated ASFV virus particles, have demonstrated limited protection in pig challenge studies due to poor immunogenicity, including weak antibody responses in some cases [36]. It has been discussed how cellular immunity plays an important role in protecting against ASFV infection and requires viral replication in the host [37], which is why nonreplicating vaccine options like killed vaccines are often ineffective. Recombinant protein-based subunit vaccines are composed of synthetic ASFV antigens and face similar challenges, and vaccine candidates in this class have only demonstrated partial protection [24]. Other options like DNA vaccines, which use plasmid DNA as carriers, and vector vaccines, which use viral or bacterial carriers, to deliver ASFV antigen genes for in vivo expression are promising options that can support CD8^+^ T cell activation, which is critical for stimulating cellular immunity [38]. However, DNA and vector vaccines still offer limited protection against ASFV infection, and it has been suggested that one of the key challenges is that currently single and even multi-antigen expression strategies do not support comprehensive immune protection, highlighting the importance of discovering more protective antigens within the ASFV genome [39].

On the other hand, LAVs have emerged as the most promising option for ASFV control and are modified versions of wild-type ASFV strains wherein one or more virulence genes that contribute to pathogenicity are deleted from the genome [40]. The safety and efficacy of these vaccines have been demonstrated in experimental testing, with protection levels reaching up to 80%–100% against homologous strains [40]. Field trials and vaccination campaigns are underway [41] but global deployment must address several issues: (1) most vaccines only work against one ASFV genotype, and it is difficult to produce a vaccine that works against multiple genotypes; (2) new strain variants may emerge due to recombination of vaccine strains with circulating strains; and (3) nonvirulent vaccine strains may acquire virulence (reversion) [42]. Other practical issues include vaccine stability because LAVs require cold-chain storage at 2–8°C [43] and have relatively short-lasting immunity of ~2–6 months [44, 45], which requires booster doses to maintain protection. These issues present logistical challenges for resource-limited settings where reliable refrigeration may not be available and in pig farming operations where revaccination could be costly and labor-intensive.

Nevertheless, there has been important commercialization progress on the vaccine development front. The first ASFV vaccines (NAVET-ASFVAC, AVAC ASF LIVE, and DACOVAC-ASF2) were recently approved for use in Vietnam. Since ASFV genotype II is endemic in Vietnam, all three vaccines are LAVs consisting of genetically modified ASFV genotype II strains with deleted virulence genes to attenuate the virus. NAVET-ASFVAC is based on the ASFV-G-ΔI177L strain, which involves the deletion of the I177L gene, a virulence-associated factor [46], while AVAC ASF LIVE utilizes the ASFV-G-ΔMGF strain, which carries deletions in multiple Multigene Family (MGF) genes that help ASFV evade the host immune system [47, 48]. DACOVAC-ASF2 is the most recently approved vaccine and is based on the ASFV-G-ΔI177L/ΔLVR strain that has the I177L gene deletion along with additional deletions in the left variable region (LVR) to further attenuate the virus [49].

Large-scale field trial testing of these vaccine strains involving over 650,000 doses in total has been performed across over 40 provinces in Vietnam [50]. The Vietnamese National Center for Veterinary Drug and Vaccine Quality Control has reported that the NAVET-ASFVAC vaccine demonstrated a 95% protective immune response when tested in over 140 farms, whereas the AVAC ASF LIVE vaccine yielded a 93.4% protective immune response when tested in over 500 farms [51]. These field trials were conducted under the regulatory supervision of the Vietnamese Ministry of Agriculture and Rural Development, and over 6 million doses of these vaccines have been produced for distribution and export [50]. It has also been reported that the DACOVAC-ASF2 vaccine provided 80%–100% protective immune responses in large-scale field trials involving ~300,000 doses, which were conducted on internal swine farms managed by the Dabaco Vietnam Group [52].

The AVAC ASF LIVE vaccine has also been exported to the Philippines, where the Philippines Department of Agriculture (DA) and Food and Drug Administration (FDA) have approved the vaccine for restricted use, with up to 150,000 doses initially [53]. The vaccine is currently being tested in field trials prior to broader potential approval [54].

The United Nations Food and Agriculture Organization (FAO) reported that the Philippines DA received 10,000 doses in August 2024 and initiated government-coordinated ASFV vaccination in the Batangas province [55]. Preliminary results from two backyard farms indicated that protective immune responses were developed in 34 out of 41 vaccinated pigs, equating to ~83% protection and motivating further expansion to other farms. Smaller-scale exports of the NAVET-ASFVAC and AVAC ASF LIVE vaccines to Nigeria and the Dominican Republic have also been initiated [42].

While these vaccine developments are encouraging, the World Organisation for Animal Health (WOAH) has also highlighted the risks of using potentially ineffective vaccine strains that may provide inadequate protection and recombination could create novel strains [56]. The WOAH recommends that vaccination should not be used as a standalone disease control method but rather as part of broader prevention and control strategies that include biosecurity measures. The United Kingdom (UK) Department for Environment, Food and Rural Affairs (Defra) has also suggested that the vaccination campaign in Vietnam has had limited success [54], as indicated by an increase in the number of ASF cases there and cited issues like the emergence of a recombinant variant strain that has features of ASFV genotype I and genotype II, which makes it difficult to achieve protection using current vaccine options [57, 58]. Indeed, it has been discussed how this new variant might replace the circulating ASFV genotype II strains in the region, leading to new challenges on the vaccine development front and potentially limiting the effectiveness of the recently approved vaccine products [58]. Such issues further highlight the importance of developing orthogonal strategies for ASFV control that complement vaccines.

### 2.2. Antiviral Drugs

While culling of ASFV-infected animals takes precedence over treatment and protective vaccines would be highly preferable [59], another ongoing research direction involves the development of broadly effective antiviral drugs to combat ASFV infections in pigs in vivo (Figure 1B). In general, the main objective is to administer an antiviral drug before infection (prophylaxis) or after infection (therapy) in order to inhibit viral genome replication, which blunts viral infection and stops the production of new virus particles. In the ASFV context, it would be advantageous to use antiviral drugs as prophylactics to prevent infection and research progress has followed this direction. Various antiviral drug candidates that inhibit ASFV replication have been described [21, 60], but there are few options with demonstrated in vivo activity.

Among them, a nucleoside analogue, cyclic cidofovir, was reported that works against ASFV strains from four different genotypes [61]. It was envisioned that prophylactic use of antiviral drugs like cyclic cidofovir might be useful for blunting outbreaks by preventing infections from spreading and could be used in coordination with widespread vaccination, especially in the early stages before vaccine-induced immunity develops. In vitro experiments showed that cyclic cidofovir has 50% inhibitory concentration (IC_50_) values around 100–500 nM against different ASFV genotypes and a selectivity index of over 120 in porcine macrophages (PAMs). It was observed that prophylactic oral administration of cyclic cidofovir at 30 mg/kg dose to ASFV-infected pigs delayed clinical symptoms and reduced viral loads in blood and tissues. The in vivo efficacy required repeated administration and depended on the dose schedule. In addition to prophylactic administration prior to virus challenge, subsequent twice-daily administration of cyclic cidofovir postinfection extended the median survival time from ~6 to ~20 days and 33% of the infected pigs in the treatment group survived until the end of the study, whereas all untreated pigs died. On the other hand, subsequent once-daily administration extended the median survival time from ~6 to ~8 days, but no pigs survived until the end of the study.

Brincidofovir is another promising antiviral drug candidate that is a kinase inhibitor, which exhibited an IC_50_ value around 3 nM against a virulent ASFV strain in vitro and a high selectivity index of over 21,000 in PAMs [62]. Prophylactic oral administration of brincidofovir at 2 mg/kg dose partially protected ASFV-infected minipigs from virus-induced mortality and histopathological damage as well as reduced viral loads in blood and various organs. Brincidofovir administration was once daily and started 7 days before virus challenge until the end of the study (day 30 postchallenge). Notably, 100% of infected minipigs without treatment died within 10 days postchallenge while 40% of minipigs in the treatment group survived until the end of the study. The other minipigs in the treatment group died within 14–16 days postinfection, demonstrating extended survival due to the treatment.

In addition, it has been reported that certain *Bacillus subtilis* probiotic strains can inhibit ASFV infection, and extracted metabolites of those strains at 1 µg/mL concentration caused a >99% reduction in ASFV titers in vitro according to viral infectivity measurements [63]. This result motivated exploration of prophylactic uses and two administration formats were tested to protect against lethal ASFV infection in pigs in vivo: (1) biologics derived from the four *B. subtilis* strains were orally administered and (2) powders derived from the four *B. subtilis* strains were mixed with pig feed. In both formats, once-daily administration was performed starting from 10 days before virus challenge until 28 days postchallenge. The prophylactic treatments prevented pig mortality whereas all infected pigs died in the untreated control groups and also reduced viral loads in blood and various organs. It was further identified that arctiin and genistein were two of the most active small-molecule metabolites found in these strains and were also tested for in vivo antiviral efficacy. Prophylactic oral administration of 2 mg/kg arctiin, 2 mg/kg genistein, or 2 mg/kg arctiin and 2 mg/kg genistein was performed following the dosing schedule described above and partially protected pigs against ASFV-induced mortality, yielding survival rates of 50%–70%. Viral loads in blood samples were reduced by up to 99% but viral loads were still detected in organs even after treatment. In summary, there are several promising antiviral drug candidates in development and their utility is focused on prophylactic applications to prevent ASFV infection, while all options require repeated dosing and more efficacious treatment outcomes are needed.

Complementing vaccine and antiviral drug development, ASFV mitigation strategies based on robust, genotype-independent virus inactivation are an important biosecurity tool and related scientific advances are readily translatable to field tests and commercialization. With growing progress in the field, mitigation is evolving from a strict focus on thermal inactivation and chemical disinfection of virus-contaminated materials in the external environment to a more multifaceted viewpoint in which functional mitigants can also be delivered to animals via feed and drinking water sources in order to inhibit virus particles in pigs, enhance immune function of healthy and infected pigs, and prevent lateral transmission from infected pigs to healthy pigs. Since mitigation strategies mainly focus on directly inactivating virus particles, it is important to first discuss the challenges of the robust ASFV structure as a mitigation target compared to other livestock viruses.

## 3. ASFV Structure as a Mitigation Target

Many livestock viruses, including ASFV, belong to the broad category of membrane-enveloped viruses, which includes viruses that possess a lipid bilayer membrane coating and pose a higher outbreak risk than nonenveloped viruses [64]. Compared to most enveloped viruses that affect livestock populations, ASFV is distinct because it is also a nucleocytoplasmic large DNA virus (NCLDV) or so-called giant virus [65]. Whereas most swine viruses, such as porcine epidemic diarrhea virus (PEDV) and porcine reproductive and respiratory syndrome virus (PRRSV), have single-stranded RNA genomes and a relatively fragile single lipid bilayer envelope coating [66, 67], ASFV has a double-stranded DNA genome and two lipid bilayer envelope coatings that bound a contiguous protein capsid. Due to these more robust features, ASFV is highly stable in biological matrices and it is generally considered more difficult to inactivate ASFV than other swine viruses [68].

Structurally, ASFV virus particles have a five-layer organization consisting of a (1) nucleoid region that contains viral DNA, (2) protein-based core shell, (3) inner lipid bilayer envelope, (4) protein capsid layer, and (5) outer lipid envelope [69]. The outer lipid envelope is only present on extracellular ASFV particles, which have around 200–300 nm diameters. In addition, the ASFV p72 protein is the main component of the capsid layer and is an important structural element that associates with the inner and outer envelopes for particle assembly [70]. From a targeting perspective, it is ideal to employ mitigation strategies that disrupt the lipid bilayer envelopes and associated viral proteins and/or damage viral nucleic acids in order to inhibit ASFV infectivity and replication processes.

## 4. Mitigation Strategies

**Figure 2:**
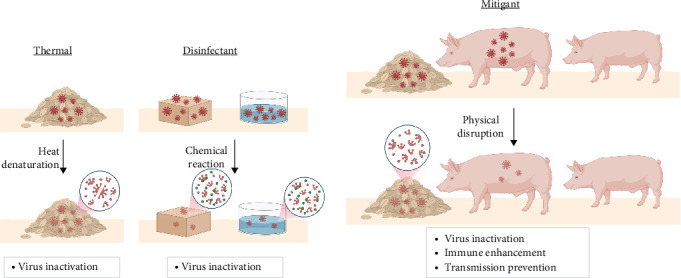
ASFV mitigation strategies. (A) Thermal treatments are useful for ASFV inactivation in feed and feed ingredients, while chemically reactive disinfectants are useful for immediate ASFV inactivation on contact surfaces and in water in some cases. (B) Physically disruptive mitigants offer a holistic healthcare viewpoint and are focused on feed or water delivery of mitigants that not only inactivate ASFV in transmission vectors like feed but also exhibit additional functionalities such as reducing clinical symptoms in infected animals, supporting immune function, and preventing lateral transmission from infected pigs to healthy pigs. Created in BioRender. Jackman, J. (2025) https://BioRender.com/3kllv4e. ASFV, African swine fever virus.

As ASFV is a major threat to pig production, extensive efforts are made to prevent infectious ASFV from reaching pig populations.  Figure 2 presents an overview of the different mitigation strategies that are used to inhibit ASFV in various settings. Thermal treatment involves storing feed and feed ingredients at elevated temperatures for short periods or at room temperature for long periods in order to provide sufficient time for degradation of potential viral contaminants (Figure 2A). The main mechanism of virus degradation involves thermally activated processes like viral protein denaturation that blunt virus infectivity. Similarly, disinfectant strategies involve the use of small-molecule chemicals with reactive properties to inactivate virus-contaminated materials that can include feed and feed ingredients as well as drinking water and fomites such as contact surfaces and worker clothing. Disinfectants typically accelerate virus particle degradation through chemical reactions or by creating a more inhospitable environment. A common theme of thermal treatment and chemical disinfectants is a primary focus on rapid inactivation of virus-contaminated materials for sanitation purposes. Building on these approaches, mitigation is a broader strategy that focuses on employing naturally occurring or synthetic molecules (“mitigants”) to not only inactivate virus particles in feed and drinking water matrices through methods of physical disruption but also deliver the mitigants to pigs in order to support positive overall health outcomes, including inhibiting virus particles during infection, reducing clinical symptoms, enhancing the immune function of animals, and preventing lateral transmission from infected pigs to healthy pigs (Figure 2B). While not every mitigant displays all of these functions, the idea of mitigation is shifting from a synonym of disinfection or inactivation to a more holistic animal health viewpoint that encompasses these additional functionalities as well.

Before introducing the latest progress and advances for each ASFV mitigation strategy, we wish to briefly cover key points related to inactivation testing that are described when discussing different testing results. Virulent ASFV strains infect primary swine monocyte and macrophage cells and cause infections in pigs [71] while nonvirulent ASFV strains have been generated that can infect commonly used cell lines such as Vero cells but do not infect primary cells [72]. There are various methods for viral titer quantification depending on the cellular system, and care must be exercised when comparing data across different assay formats [73]. Viral infection levels can also be monitored in terms of relative nucleic acid levels by PCR and conformationally intact antigen levels by ELISA. The p72 protein is often the antigen of choice because it is the key one for detecting ASFV infection [74]. There are also in vivo pig bioassays, whereby an ASFV-contaminated sample is treated with a mitigant and then fed to pigs to determine if complete inactivation occurred. Such capabilities provide useful tools to characterize different types of ASFV mitigation strategies and are discussed throughout this section.

### 4.1. Thermal Inactivation

ASFV is highly stable and can remain infectious in blood and other biological fluids for weeks, months, or even years (see ref. [75] and references therein). This issue has been extensively discussed before and has led to detailed exploration of virus persistence in different environmental settings. One of the most important parameters that affect virus stability is temperature, which can cause denaturation of viral structural components such as envelope proteins [76]. Related studies have covered temperature-dependent viral persistence in natural environmental settings as well as the development of strategies to thermally inactivate ASFV contaminants in matrices such as feed and feed ingredients by applying elevated temperatures (>50°C).

#### 4.1.1. Temperature Stability

We begin by covering recent thermal stability studies that describe how natural temperature variations affect viral persistence in different settings such as animal carcasses and tissues, biological matrices, fomite surfaces, and feed materials. It has been reported that skin samples from ASFV-infected pigs and wild boars can remain infectious at room temperature for up to 3 months [75]. Kinetic analysis of ASFV contamination in pig tissues supports that the half-lives of viral infectivity vary from ~32 days at −20°C to ~9 h at +23°C [77]. Higher moisture content and the presence of organic matter also tended to accelerate ASFV inactivation in the tissue samples. These effects were attributed to greater tissue preservation in matrices with low moisture content (e.g., straw, hay), while organic matter such as water, soil, and leaf litter caused quicker virus inactivation, which may be related to environmental pH effects or the types of reactive molecules present in the organic matter. Similar temperature-dependent results have been observed for ASFV contamination in soil samples, with infectious virus detectable after 112 and 42 days incubation at +4 and +22°C, respectively [78]. In addition, ASFV-infected blood samples from an infected wild boar were incubated in different types of soil and high stability was observed in sandy soils but no infectious virus remained in acidic forest soils [79].

Another study reported that the half-life of ASFV at 4°C in serum-containing media was around 36–119 days and the half-life decreased to around 1 day at 37°C and to around 1 min at 60°C [80]. Interestingly, PCR testing has demonstrated that ASFV nucleic acid levels remain largely stable for at least 5 days at temperatures up to 70°C, whereas ELISA results showed temperature-dependent loss of ASFV antigen conformation after 56°C treatment for 1 h [81]. This finding supports that the loss of viral infectivity mainly stems from structural damage to the virus particle (e.g., protein conformational changes). A combination of moderately elevated temperature (48°C), alkaline conditions (pH 10.2), and high hydrogen peroxide concentration (103 mM) has also been reported to inhibit ASFV infectivity in porcine plasma [82]. Separately, groundwater contamination studies have shown that ASFV at 4°C can remain infectious in river water for greater than 42 days but is rendered noninfective after 28 and 14 days at 15 and 21°C, respectively [83].

Additionally, the persistence of dried infectious ASFV on porous and non-porous surfaces such as glass, metal, rubber, and cellulose paper was investigated at different environmental temperatures between 25 and 42°C [84]. In this case, ASFV inactivation kinetics were analyzed by measuring the decimal reduction time (*D*_T_), which is defined as the time required to reduce ASFV infectivity per 1 log at a certain temperature (T). A smaller *D*_T_ value means quicker inactivation. A similar trend was observed on all surfaces whereby higher temperature led to quicker inactivation and the corresponding *D*_25_, *D*_33_, and *D*_42_ values were around 1.42–2.42, 0.72–1.94, and 0.07–0.23 days, respectively.

The persistence of infectious ASFV in these different environments has prompted detailed examination of ASFV infectivity in feed and feed ingredients, which is an important transmission risk for livestock viruses in general. By mimicking storage conditions associated with transoceanic shipping over 30 days, it was determined that infectious ASFV remained viable in 9 out of 11 feed ingredients and had a half-life of ~1–2 days [85]. A more detailed analysis of ASFV stability in the different feed ingredients over a 30-day simulated shipping period provided a revised estimate that infectious ASFV has a half-life of around 9–14 days [86]. In another study, ASFV was incubated in complete feed, soybean meal, and ground corncobs at 4.4, 20, or 35°C for up to 1 year, and it was identified that viral infectivity was maintained in soybean meal at 4.4°C for at least 112 days, at 20°C for 21 days, and at 35°C for 7 days [87]. These results demonstrate that ASFV is more sensitive to higher temperatures and have prompted the development of thermal inactivation strategies based on elevated temperature treatment.

#### 4.1.2. Thermal Treatment

While extended storage of feed and feed ingredients can help to reduce contamination risk, thermal treatment of these materials at elevated temperatures is a complementary strategy that provides quicker and more robust inactivation. For this reason, several recent studies have investigated how elevated temperatures affect ASFV infectivity in complete feed, feed ingredients, crops, and swill, which has also provided scientific evidence to guide regulatory policy developments. These studies are summarized in Table 1 and discussed below.

The combined effect of drying and heat treatment has been reported, whereby ASFV-contaminated field crops—a potential source of material for feed and bedding—were dried for 2 h at room temperature and then incubated for 1 h at elevated temperatures ranging between 40 and 75°C [88]. The drying step reduced viral infectivity of ASFV-infected field crops in cell-based assays to undetectable levels, even at room temperature. However, compared to drying alone, ASFV viral nucleic acids were more degraded but still present after subsequent thermal treatment according to PCR testing. Additional testing with blood samples demonstrated loss of viral infectivity at ≥55°C, supporting that elevated temperatures in that range are most effective at viral inactivation in biologically relevant matrices. The use of thermal treatment at elevated temperatures (≥55°C) is important to ensure robust ASFV inactivation because lack of viral infectivity in cell-based assays may be due to insufficient assay sensitivity and infectious ASFV residues can still persist in some cases. For example, it has been reported that dry-cured meat products from ASFV-infected pigs were noninfective according to cell-based assays but still caused infection in healthy pigs that consumed the meat products [94].

For thermal inactivation of ASFV in feed ingredients such as meat and bone meal, soybean meal, and maize grain, the corresponding *D*_60_, *D*_70_, *D*_80_, and *D*_85_ values were around 5–7, 2–3, 1–2, and 0.2–1 min, respectively [89]. Another study reported that a significant reduction in ASFV infectivity in three different swill formula occurred starting at 60°C and the corresponding *D*_60_, *D*_70_, *D*_75_, and *D*_80_ values were around 23–33, 6–11, ~2, and ~1 min, respectively [90]. Importantly, based on analyzing the viral inactivation kinetics, it was concluded that complete inactivation of ASFV contamination in swill could require up to 119 and 4 min at 70 and 90°C, respectively, which is important for guiding policy recommendations that are further discussed below. Another recent study reported that infectious ASFV can remain viable in porcine serum for >60 days at temperatures up to 37°C, 2 days at 50°C, 1 day at 60°C, and ≤5 min at 70°C, which supports a similar trend of temperature-dependent ASFV inactivation [91]. As discussed above, the presence of organic matter such as feed, straw, or wood shavings also accelerated virus degradation, which was attributed to acidic conditions and reactive chemicals within the materials.

Such findings fit well with ASFV-related regulatory guidelines regarding the import of plant-based feed ingredients for use in livestock feed [95]. The Canadian Food Inspection Agency (CFIA) has mandated that, for imports from regions not recognized as free of ASF, thermal treatment must be performed whereby the product temperature reaches at least 70°C for 30 min or 85°C for 5 min in order to ensure sufficient virus inactivation. Alternatively, the imported feed ingredients can be stored for at least 20 days at 20°C or 100 days at 10°C. More broadly, the United States Department of Agriculture (USDA) published an ASF Response Plan that describes how heat application is an effective strategy for cleaning and disinfection as part of biosecurity efforts and is also useful in disease response contexts [96]. The plan cited WOAH recommendations that effective ASFV inactivation requires thermal treatment at 56°C for 70 min or at 60°C for 20 min.

The WOAH has also issued detailed guidelines advising that ASFV inactivation in swill requires thermal treatment at a minimum of 90°C for at least 60 min or 121°C for at least 10 min, whereas ASFV inactivation in meat products requires a minimum temperature of 70°C throughout the meat for at least 30 min, and ASFV inactivation in pig litter or manure requires a minimum temperature of 55°C for at least 60 min or a minimum temperature of 70°C for at least 30 min [97]. The FAO has more generally recommended that effective ASFV inactivation in porcine-related materials (e.g., meat products, carcasses, blood, abattoir swill) can be achieved by incubating at 70°C for 30 min [98].

The Japanese Ministry of Agriculture, Forestry and Fisheries (MAFF) has also advised that food waste-derived swill must be treated at 90°C for at least 60 min [99] in line with the WOAH guidelines. Conversely, the South Korean Ministry of Agriculture, Food and Rural Affairs (MAFRA) only requires thermal treatment at 80°C for 30 min, which is below the WOAH recommendations, and it has been suggested that these mandated treatment conditions are potentially insufficient for effective ASFV inactivation [100]. This deviation raises concerns about incomplete ASFV inactivation, potentially increasing transmission risks and highlighting the importance of formulating evidence-backed biosecurity practices. While this practical example demonstrates that there can be some quantitative differences in recommended thermal treatment conditions depending on the regulatory agency, it is generally recognized that thermal treatment at >55°C is effective for ASFV inactivation. Combined with the latest scientific evidence described above, the overall body of scientific evidence supports that thermal treatment in the >70°C range is effective in most cases, while swill processing demands more stringent conditions for precaution.

There has also been extensive interest in preventing ASFV contamination of spray-dried animal plasma (SDAP) products, including spray-dried porcine plasma (SDPP), which are popular feed ingredients for pig production. SDAP manufacturing involves a short (< 1 s) spray-drying step at 80°C outlet temperature, and it was found that this step causes an ~2 log_10_ drop in ASFV infectivity [92]. Furthermore, to mimic the longer residence times found in commercial spray dryers, an extended incubation (1 min) at 80°C after drying was found to decrease ASFV infectivity by over a 4 log_10_ drop, which is considered suitable for effective ASFV inactivation. Another study investigated the effects of spray drying on ASFV infectivity in SDPP samples with the outlet temperature set at 80 or 71°C and a 3.2–4.2 log_10_ or 2.5–2.8 log_10_ drop in ASFV infectivity was achieved at the two respective temperatures [93]. After a subsequent 14-day storage period at 4 or 20°C, complete inactivation of all ASFV-contaminated SDPP samples was achieved and no infectious ASFV could be detected. In addition, a quantitative risk assessment model was applied to SDPP processing of ASFV-contaminated plasma and supported the feasibility of ensuring ASFV decontamination in large batches up to 20 tons [101].

While thermal inactivation strategies play an important role in combating potential ASFV contamination of feed and feed ingredients, these strategies are typically one-time treatments and re-exposure to ASFV after treatment is possible. For example, the postmanufacturing exposure of SDPP to infectious ASFV has been investigated and room temperature storage of the ASFV-contaminated SDPP for 1 or 2 weeks led to >2.8 log_10_ and >5.7 log_10_ drops in viral infectivity, respectively, indicating that complete inactivation can be achieved in 2 weeks [102]. On the other hand, storage at 4°C was insufficient to inactivate ASFV even after 5 weeks. This issue has also been recently raised in the context of persistent ASFV contamination in a pilot-scale feed mill facility [103] and points to the utility of employing additional inactivation strategies, especially fast-acting ones that support repeated use.

### 4.2. Disinfectants

Another widely used strategy for ASFV decontamination involves the use of chemically reactive disinfectants to inactivate infectious ASFV that may be present on contaminated materials such as vehicles, equipment, clothing, and tools within farm settings [28]. Contaminated materials have been implicated in introducing ASFV to farms, and disinfectants work by inhibiting ASFV by up to >4 log_10_ level, which significantly reduces transmission risk on farms. Compared to thermal inactivation, chemical disinfectants are more amenable to repeated use in order to minimize chances of re-exposure. While many different types of disinfectants exist for various applications, due consideration of the appropriate disinfectant is important. For example, different types of acids and bases can inactivate viruses by causing pH changes that affect viral protein conformations and overall particle stability but may also be corrosive and irritating to contact surfaces [104]. Other disinfectants like aldehydes damage viral proteins and nucleic acids through alkylation reactions, whereas oxidizing disinfectants can damage viral proteins, disrupt the lipid bilayer envelope, and oxidize nucleic acids.

Regardless of the mechanism, disinfectants exhibit antiviral properties against ASFV by displaying virucidal activity, which means that disinfectants directly inhibit ASFV particles. Experimentally, the common experimental format is to incubate virus particles with a disinfectant for a defined time span before adding the virus–disinfectant mixture to cells to measure residual infectivity. If a disinfectant is active, it will inhibit the virus and prevent cell infection, and the generally accepted performance cutoff for a highly active disinfectant is a 99.99% (4 log_10_) drop in viral infectivity, while some studies consider a 99.9% (3 log_10_) drop to be effective.

Early studies on ASFV disinfectants focus on preventing contamination of steel and plastic surfaces, and it was observed that 500–1000 ppm sodium hypochlorite and 1% citric acid were effective at complete ASFV disinfection by 4 log_10_ units, whereas 4% sodium carbonate inhibited ASFV by 3 log_10_ units [105]. It was later found that 2000 ppm sodium hypochlorite and 2% citric acid were also effective at inhibiting ASFV on porous wood surfaces by 4 log_10_ units [106]. Disinfection efficiency also depends on the medium; both sodium hypochlorite and citric acid treatment of steel surfaces were less effective at inhibiting ASFV in the presence of fresh swine blood, and only citric acid was active in swine feces [107]. These surface decontamination results offer insight into the efficiency of selected disinfectants against ASFV and have led to the development of virucidal suspension tests for more systematic evaluation of various disinfectant candidates.

These efforts have led to rigorous assessment of ASFV disinfectants and are motivated by the shift from using disinfectants that are known to be widely active against enveloped viruses but had not yet been tested against ASFV to validating certain disinfectants against ASFV for reliable use. With limited ASFV-specific data until recently, most early disinfectant options were considered based on known efficacy against other membrane-enveloped viruses relevant to livestock applications [30]. For example, one study suggested disinfectants that might be useful for combating ASFV in the UK setting by evaluating virucidal efficacy data against related viruses and considering which of the promising disinfectants were already approved for use there [23]. Options include formaldehyde, sodium hypochlorite, sodium hydroxide, glutaraldehyde, phenols, organic acids, hydrogen peroxide, potassium peroxymonosulfate, and quaternary ammonium compounds. Ongoing efforts to test the virucidal properties of these disinfectants and other candidates against ASFV are guiding targeted usage and providing a scientific foundation to support policy recommendations. A summary of this research progress is covered in Table 2 and discussed below.

#### 4.2.1. Comparison Studies

Many studies have comparatively investigated disinfectant candidate panels in aqueous suspensions, and we first cover these results before commenting on more specific examples. In one study, the antiviral effects of four commercial disinfectants—comprising sodium hypochlorite, potassium peroxymonosulfate, glutaraldehyde, or quaternary ammonium compounds as the active ingredient—on the infectivity of the cell-culture-adapted, nonvirulent ASFV BA71V strain was investigated using Vero cells [108]. Products based on glutaraldehyde or quaternary ammonium compounds showed high cytotoxicity to the Vero cells, which precluded further examination. Conversely, products based on sodium hypochlorite and potassium peroxymonosulfate demonstrated more favorable virucidal properties. The sodium hypochlorite-based product was active at 0.5% and 1% concentrations and caused ASFV infectivity drops of >4 log_10_ but was less effective in the presence of soil-mimicking organic compounds. On the other hand, the potassium peroxymonosulfate-based product at 1% concentration was more effective in highly soiled conditions but was cytotoxic in standard conditions. A follow-up study with a modified protocol to enable cytotoxic compound evaluation was performed to test the antiviral activity of eight active compounds—formaldehyde, sodium hypochlorite, caustic soda, glutaraldehyde, phenol, benzalkonium chloride, potassium peroxymonosulfate, and acetic acid—and it was reported that sodium hypochlorite, caustic soda, and potassium peroxymonosulfate had the best inactivation performance [109]. By contrast, benzalkonium chloride, which is a quaternary ammonium compound, glutaraldehyde, and formaldehyde also exhibited virucidal properties but had high cytotoxicity.

Another recent study reported testing a panel of 24 commercial disinfectants against the ASFV BA71V strain and included compounds categorized as oxidizing agents, acids, aldehydes, formic acids, and phenols [110]. It was found that disinfectants based on formic acid, phenolic compounds, and oxidizing agents decreased ASFV infectivity by >4 log_10_, whereas other disinfectants based on hydrogen peroxide, aldehyde, and quaternary ammonium compounds also inhibited ASFV to varying extents but had high cytotoxicity. These results are consistent with the reported efficacy of oxidizing agents such as sodium hypochlorite and potassium peroxymonosulfate described in the aforementioned studies and other oxidizing agents such as chlorine dioxide [111] and hydrogen peroxide [81] have also been reported to inhibit ASFV. In addition, another oxidizing agent, iodine, has been reported to have high inactivation efficiency in a complexed form [112] and works synergistically with organic acids [113]. The results of the panel study further support that aldehydes like glutaraldehyde are effective at ASFV inactivation but are cytotoxic. Even so, it should also be noted that certain ones, especially formaldehyde, are still being explored additionally as mitigants for ASFV inactivation in more complex biomatrices such as feed and display rapid inactivation kinetics in suspension tests [114].

Several recent studies have also evaluated disinfectants against virulent ASFV strains using PAM cells. For example, it has been reported that various disinfectants—an iodine and acid mixed solution, potassium peroxymonosulfate, citric acid, sodium dichloroisocyanurate, glutaraldehyde/deciquam, and deciquam—wereeffective at inactivating ASFV in immersion and spraying formats [115]. However, in the presence of organic matter, the disinfectants were appreciably less active, indicating they are best suited for decontaminating clean surfaces rather than mitigation in biomatrices. Interestingly, it has also been reported that a mixture of quaternary ammonium compounds inactivates ASFV better than potassium hydrogen peroxymonosulfate, suggesting that different ASFV strains may have varying susceptibilities and also that compositions of different molecule types can be tailored to improve virucidal performance [116]. In terms of applications testing, four commercial disinfectants—composed of a cationic surfactant, glutaraldehyde + cationic/anionic surfactants, iodine, and glutaraldehyde + cationic surfactant – were tested in the absence of interfering organic species and were all found to rapidly reduce virulent ASFV infectivity by >4 log_10_ within 15 min [117]. Another study tested five commercial disinfectants comprising glutaraldehyde or quaternary ammonium compounds as the active ingredient and noted that all had similarly high antiviral activity against a virulent ASFV strain, while it was further reported that these disinfectants had high cytotoxicity that likely restricts their use to surface decontamination applications [118].

One recent study further demonstrated that sodium hypochlorite, glutaraldehyde, potassium peroxysulfate, sodium hydroxide, phenol, and formaldehyde maintain ASFV inactivation efficacy across a wide range of temperatures from +21 to −20°C [119]. Conversely, benzalkonium chloride and acetic acid were less effective at sub-zero temperatures, and these findings provide guidance regarding which disinfectants would be useful for surface decontamination in cold environments.

A practical example of a disinfectant application in an ASF outbreak setting was described for a medium-sized farm in Uganda [125]. After the outbreak started, workers rinsed their boots in a water bath when moving from the pig slaughter facilities to the pig pens. The active ingredient in the water bath was described as a commercial disinfectant containing ammonium chloride, which likely refers to a quaternary ammonium compound although the exact formulation was not specified. While boot rinsing was performed, it was noted that the boots were not physically cleaned prior to immersion. As such, organic material was likely still present on the boots, which would limit ASFV inactivation efficacy as discussed above. This example illustrates both the feasibility of using quaternary ammonium compounds for surface decontamination and the need for improved biosecurity practices, such as removing organic material before disinfection rinsing, to enhance efficacy.

These findings have also provided a scientific foundation to support regulatory decisions regarding the use of particular disinfectants for ASF biosecurity. The USDA Animal and Plant Health Inspection Service (APHIS) has approved several disinfectants for emergency use against ASFV, including those containing citric acid, acetic acid, sodium hypochlorite, and thymol, for surface decontamination [126]. Other active ingredients found in disinfectants approved by the USDA APHIS for use against ASFV in farm settings include potassium peroxymonosulfate, several quaternary ammonium compounds, and oxidizing agents. Interestingly, the oxidizing agent sodium dichloro-striazinetrione that releases hypochlorous acid is approved for use in disinfection baths for boots, while the listed quaternary ammonium compounds are not described for this application [127].

The WOAH has also provided recommendations that include sodium hydroxide, hypochlorites, formaldehyde, and oxidizing iodine compounds while noting that virucidal efficacy can vary depending on pH, incubation time, and the presence of organic matter [128]. In some cases, disinfectants can be used for soil decontamination as well, and there are affordable options such as lime products based on calcium hydroxide, which can be ~20 times cheaper than surfactant options [129]. At the same time, there has been discussion about whether applying basic disinfectants to acidic soils is appropriate, whereas acidic disinfectants like citric acid might be more useful [79]. In addition to these cost and compatibility factors, other practical considerations for disinfectant selection include handling safety, corrosiveness, chemical stability, and efficacy in the presence of organic matter.

Overall, these comparison studies have also highlighted the importance of developing disinfectants that balance high virucidal activity with low cytotoxicity for broad usage and have prompted efforts to find more natural and sustainable disinfectant options. Recently, a powdered disinfectant containing thymol as the active ingredient was also reported to effectively inhibit ASFV [130] and highlights the push towards more natural and sustainable disinfectant options. Within this scope, we introduce two examples of natural disinfectants that are being considered for ASFV disinfectant applications.

#### 4.2.2. Water

Oxidizing agents are one of the most effective types of disinfectants to inactivate ASFV, although many compounds in this class are synthetic and not ideal for uses involving human or animal contact. As such, there is high interest in developing safer, natural versions for broader application usage. Ozonized water is a promising candidate that can be produced at >40 mg/L concentrations by simple electrolysis of water and contains ozone molecules in the water. Ozone is a nontoxic oxidizing agent that can rapidly inactivate viruses and bacteria and safely decomposes into oxygen within a few days. It was recently demonstrated that 1-min treatment with 5–20 mg/L ozonized water could reduce virulent ASFV infectivity by up to 3 log_10_ (99.9%) in a dose-dependent manner in PAM cell experiments [120].

In addition, there has been exploration of chlorinated water for inactivating ASFV, which involves electrolysis of acidic water to generate the HOCl oxidizing agent [121]. Electrolyzed water containing 40–60 ppm free chlorine was effective at reducing ASFV infectivity by >4 log_10_ whereas lower concentrations had appreciably less activity. In water containing 5% fetal bovine serum (FBS) to mimic soil conditions, effective ASFV inactivation of 4 log_10_ or greater required at least 80–100 ppm free chlorine in the water. These findings support that chlorinated water could be useful for decontaminating contact surfaces and soil and may also be used in drinking water.

#### 4.2.3. Natural Plant Extracts

Inspired by plants used in African traditional medicine, 28 plant extracts from various natural sources were tested for ASFV disinfectant properties [122]. Four extracts inhibited ASFV replication in infected cells by >80% (i.e., antiviral), while six different extracts directly inhibited ASFV virus particles by >80% (i.e., virucidal). These findings support that extracts can have different mechanisms of antiviral activity and an extract from the *Sarcocephalus latifolius* root, which has been found to contain medicinally active quinovic acid glycosides and monoterpene indole alkaloids [131], had the highest inactivating effect on ASFV infectivity (~100% drop).

Anecdotally, there have also been stories about how rural farmers use plants for ASFV disease management, including unsubstantiated reports that feeding *Ancistrocladus uncinatus* plant preparations to pigs helps ward off ASF disease [123]. This background context motivated researchers to extract compounds from the leaf, root, and stem portions of the plant, which included alkaloids, glycosides, steroids, saponins, flavonoids, and tannins, and explore how the extracts might be used as disinfectants. At ~5 mg/mL concentration, the crude acetone extract was cytotoxic to a primary bone marrow culture (PBMC), while lower extract concentrations in the range of 0.0078–1 mg/mL concentration were subsequently tested in antiviral assays. Extracts prepared using different organic solvents were tested for inhibitory activity against a virulent ASFV strain from Nigeria by using a PBMC cell culture system, and it was discovered that the acetone, dichloromethane, and methanol extracts had potent inhibitory activities, which were confirmed by PCR experiments. On the other hand, the hexane and chloroform extracts had weaker antiviral effects. These results support the validity that plant extracts can inhibit ASFV according to antiviral and/or virucidal mechanisms, while high cytotoxicity was noted and solubility improvements are needed.

A more recent study investigated 14 plant extracts and found that only peppermint extract at 1.05% concentration was effective at reducing ASFV infectivity by >4 log_10_ while maintaining acceptable cell viability [124]. Other test materials like strawberry and raspberry extracts are known to be active against other viruses [132] but were not effective against ASFV, emphasizing the challenges of inactivating its complex envelope structure. In general, it was also noted that organic contaminants representative of soil conditions tended to decrease the antiviral activity of plant extracts, and it was discussed how effective use of plant extracts as disinfectants should be preceded by pre-cleaning contact surfaces to physically remove organic debris.

Collectively, these findings indicate that a wide range of synthetic and natural disinfectants exhibit high ASFV inactivation properties with fast-acting mechanisms and are important components of biosecurity efforts. It should be emphasized that disinfectants work optimally on pre-cleaned surfaces devoid of organic contaminants. On the other hand, most disinfectants, even natural ones, have relatively high cell cytotoxicity levels or other disadvantageous properties like causing irritation or corrosion and are generally not ideal for mitigation in more complex biomatrices like feed due to safety and efficacy issues. While disinfectants will continue to play a key role in ASFV biosecurity, these limitations have also increased attention to develop mitigants, which are generally classified as physically disruptive antiviral molecules that are designed to inhibit ASFV in more complex biological matrices such as feed as well as exhibit a broader set of health-promoting functions.

### 4.3. Mitigants

Reports describing the development of antiviral molecules that directly inhibit ASFV and other swine viruses according to virucidal mechanisms often use the terms disinfectant and mitigant interchangeably, but it is useful to make a distinction between these two terms in order to critically analyze the latest progress in the field. As explained above, disinfectants are focused on immediate decontamination of virus-laden surfaces as judged by virus infectivity drops and work well on pre-cleaned surfaces but are generally not targeted for use in more complex biomatrices such as feed. Due to toxicities and irritability related to their chemical reactivity, disinfectants are usually precluded from application uses involving animal or human contact. On the other hand, mitigants are antiviral molecules that not only inhibit ASFV in transmission vectors such as feed and water but are also delivered to animals directly and may exhibit positive health benefits such as in vivo antiviral activity, immune enhancement, and prevention of lateral transmission from infected pigs to healthy pigs. For example, theoretical analysis has shown how feed mitigants can reduce the infection probability of a pig that consumes ASFV-contaminated feed [133]. In many cases, the mitigants being used are from natural sources and have favorable biocompatibility based on causing physical disruption of enveloped viruses without inducing chemical reactions, while there is still some competition from repurposed disinfectants such as formaldehyde.  Table 3 presents a comprehensive overview of the latest ASFV mitigation studies, and we cover these results in this section in order to introduce different classes of promising mitigants.

#### 4.3.1. Monoglycerides

Widely found in natural sources such as tropical oils and milk, monoglycerides (MGs) are nonionic molecules that are approved by regulatory agencies for food and agricultural applications [145, 146]. They have long been studied as broad-spectrum antimicrobial agents that can inhibit membrane-enveloped viral and bacterial pathogens [147]. It has been reported that medium-chain MGs with saturated hydrocarbon tails, especially 12-carbon-long glycerol monolaurate (GML), have potent inhibitory activity against enveloped viruses such as human immunodeficiency virus (HIV) and herpes simplex virus (HSV) [148]. MGs exhibit antiviral properties by physically disrupting the lipid bilayer surrounding enveloped virus particles [149] and can also exhibit additional mechanisms of antiviral activity, such as stabilizing cellular membranes to prevent infectious pathogen entry [150] and influencing cellular signaling networks [151]. Furthermore, certain MGs exhibit anti-inflammatory properties as well [152–154]. All of these mechanisms stem from the physical interaction of MGs with phospholipid membranes, which is distinct from the chemical reactivity of typical disinfectants.

In the ASFV context, the antiviral activity of GML has been explored in liquid and feed environments [134]. The non-virulent ASFV BA71V strain was used with Vero cells, and initial virucidal experiments in aqueous buffer conditions identified that GML was more effective at reducing ASFV infectivity than equivalent concentrations of different medium-chain fatty acids (MCFAs), including 8-carbon-long caprylic acid, 10-carbon-long capric acid, and 12-carbon-long lauric acid, and GML caused an ~98% drop in viral infectivity. Additional experiments revealed that GML exhibited multiple mechanisms of antiviral activity, leading to an overall 99.8% reduction in viral infectivity. Mitigation experiments in feed showed that GML reduced ASFV infectivity in a dose-dependent manner, and a 2 wt% inclusion rate caused an 88% drop in viral infectivity after 30-min incubation. The inclusion of 2 wt% GML markedly accelerated ASFV mitigation overall, leading to a 98% drop in viral infectivity compared to the initial condition. By contrast, a free-flowing, dry MCFA blend containing silica carrier had no effect on ASFV infectivity. More detailed analysis of the feed samples showed that GML did not affect ASFV DNA levels measured by PCR but did affect ASFV p72 capsid protein conformational properties according to ELISA measurements (consistent with reported effects of GML on altering the conformation of membrane-associated structural proteins of other viruses [155]), supporting that GML mitigation damages ASFV particle integrity but does not affect viral nucleic acids.

Follow-up studies have verified that GML can also inhibit a virulent, wild-type ASFV strain (Armenia/07) and mitigate infection of PAM cells in vitro [156]. Highly active GML concentrations had only minor effects on PAM cell viability, supporting that GML is potentially useful to inhibit ASFV infection in pigs. A solid-phase blend of GML and sodium diformate, which is a known antimicrobial compound [157], has also been reported to inhibit ASFV infectivity in feed [135]. In that study, a longer time scale of feed mitigation was evaluated whereby the feed additive at a 0.3 wt% inclusion rate inhibited ASFV infectivity by 82%, 92%, 99%, and 100% within <30 min, 1 day, 3 days, and 7 days postincubation, respectively.

Recently, there has also been exploration of a liquid-phase mitigant mixture composed of GML and medium-chain lactylates to inhibit ASFV and other enveloped viruses such as influenza A virus (IAV) [136]. The mixture could be added directly to aqueous solution, and 1:50 dilutions upward demonstrated virucidal activity against ASFV and IAV based on viral infectivity measurements. Notably, for the ASFV case, the virucidal doses also caused reduced levels of intact viral nucleic acids according to PCR experiments, which is consistent with virus particle disruption and was further supported by measured changes in membrane ionic permeability. The mixture also maintained cell viability at concentrations up to 1:10 dilution, supporting that there is an effective dose range that inhibits ASFV and is non-cytotoxic. In terms of potential utility, the mixture could be added to drinking water lines for rapid response to ASFV outbreaks, while it was also shown to be effective at mitigating ASFV in feed.

Together, these findings support that GML is capable of mitigating ASFV infectivity in liquid and feed conditions while calling for further evaluation of potential antiviral effects in animal models. These possibilities are supported by recent findings that orally administered GML can reduce mortality, clinical symptoms, viral loads, and inflammation in PEDV-infected piglets [158] and can also reduce clinical symptoms, viral loads, and inflammation in Seneca Valley virus (SVV)-infected piglets [159]. While GML has been the focus of ASFV mitigation studies, other MGs, especially 8-carbon-long glycerol monocaprylate and 10-carbon-long glycerol monocaprate, have been reported to effectively inhibit PEDV and PRRSV in feed mitigation and in vivo therapeutic contexts [160, 161] and merit further attention for ASFV applications.

#### 4.3.2. MCFAs

MCFAs are naturally occurring lipid amphiphiles that have a single hydrocarbon chain and are also widely approved for food and agricultural applications [145]. Defined as fatty acids with 6- to 12-carbon-long chains, MCFAs are a key building block of MGs and exhibit membrane-disruptive properties against various types of membrane-enveloped viruses and bacteria [162, 163]. Past studies have shown that 10- and 12-carbon-long fatty acids called capric acid and lauric acid, respectively, are highly active and corresponding 6- and 8-carbon-long fatty acid chains called caproic and caprylic acids also have advantages such as liquid phase properties around room temperature [164].

Inspired by past efforts at PEDV mitigation [165, 166], early work on testing MCFAs against ASFV was done in comparison to formaldehyde. The antiviral activity of an MCFA blend composed of caproic acid, caprylic acid, and capric acid in a 1:1:1 ratio was tested along with a 37% aqueous formaldehyde and propionic acid mixture and involved measuring mitigation effects on ASFV BA71V strain infectivity in a Vero cell model [137]. The MCFA blend reduced ASFV infectivity by around 82% at concentrations as low as 0.13%, and 0.6% MCFA caused >99.9% reduction. At higher MCFA concentrations, ASFV infectivity was reduced below limits of detection. Similar trends were observed with formaldehyde, which was effective at reducing ASFV infectivity by around 82% at concentrations as low as 0.03% and caused >99.9% inhibition at 0.3% concentration. Adjusting for the different molecular weights of the two mitigants, the data support that MCFA had similar, if not better, performance to formaldehyde in terms of molar concentrations.

In the same study, various feed ingredients were then spiked with the virulent ASFV Georgia 2007 strain and stored for 30 days according to transoceanic shipping conditions. The feed samples were pre- or posttreated with 1% MCFA or 0.33% formaldehyde and infected ASFV was not detected in any treated sample according to PAM cell infection experiments. Interestingly, however, ASFV nucleic acid levels remained unchanged in the MCFA-treated feed samples, whereas formaldehyde treatment caused marked reductions in some feed ingredients. The lack of nucleic acid damage caused by MCFA agrees well with GML mitigation data, supporting that membrane-disruptive mitigants do not necessarily damage viral nucleic acids in the ASFV case. The feed samples were then fed to pigs in a bioassay format [167] and no pigs exhibited clinical symptoms of ASFV infection. Out of the 24 pigs in the treatment groups, only one pig in the MCFA treatment group acquired ASFV infection based on virological testing after euthanasia.

There has also been interest in exploring how the antiviral activity of different MCFA mixtures compares, which has led researchers to explore three compositions consisting of (1) caprylic acid alone, (2) a 1:1:1 mixture of caproic acid, caprylic acid, and capric acid, and (3) a 1:1:1 mixture of caprylic acid, capric acid, and lauric acid [138]. All candidate formulations maintained high PAM cell viability at 100 µg/mL mitigant concentrations whereas mixture (2) was more cytotoxic at 200 µg/mL concentration. The different MCFA mixtures were added to complete feed at inclusion rates of 0.125, 0.25, 0.375, or 0.5 wt% and inhibitory effects on the virulent ASFV Pig/Hanoi/2019/01 strain were investigated. According to PCR data, all mixtures exhibited dose-dependent inhibitory effects in feed. Among them, mixture (2) was the best-performing one and caused a decrease in ASFV nucleic acid levels at mitigant inclusion rates as low as 0.125%, whereas mixtures (1) and (3) were active starting from inclusion rates of 0.375 and 0.25 wt%, respectively. However, none of the mixtures affected ASFV infectivity in a PAM cell culture system based on viral titer measurements.

#### 4.3.3. Formaldehyde

Formaldehyde is industrially produced at large scales and is widely used as an antimicrobial disinfectant and additive in agricultural applications. Formaldehyde has been reported to inhibit a wide range of enveloped viruses and mainly works by crosslinking viral proteins and nucleic acids [168, 169]. This mechanism has contributed to the role of formaldehyde and other mechanistically similar aldehydes as chemically reactive disinfectants and their established use in surface decontamination applications has led to exploring broader use in feed decontamination. While formaldehyde is still widely used in major markets worldwide, there is growing recognition of its carcinogenic risks [170] and some regulatory jurisdictions like the European Union have restricted its use in applications such as pathogen feed mitigation [171]. Nevertheless, due to its perceived high antiviral efficacy and low cost, formaldehyde is still widely explored for inhibiting swine viruses such as PEDV in feed [172], and recent studies have focused on evaluating its ASFV mitigation properties. The direct comparison with MCFA is reported above, and this section focuses on other formaldehyde-based products.

In one study, 3 kg/ton inclusion rates of different formaldehyde-based products (2 liquid and 1 dry) were evaluated for ASFV feed mitigation. Using the virulent ASFV VN/Pig/HN/19 strain, PCR measurements were performed to determine ASFV nucleic acid levels at 1, 3, or 7 days postcontamination and one liquid product reduced nucleic acid levels after only 1 day whereas the other two products acted more slowly [139]. The top-performing product was also effective at reducing ASFV infectivity in feed by day 3 postcontamination according to viral titration measurements in PAM cells and the maximum effect resulted in a 79% drop in viral infectivity. The dry product also reduced viral infectivity to a smaller extent by day 7 postcontamination while the other liquid product was ineffective at reducing viral infectivity.

Another formaldehyde-based product was also evaluated as a feed mitigant to inhibit the virulent ASFV VN/Pig/Hue/1270 strain [140]. Different inclusion rates of 1, 2, or 3 kg/ton were tested, and PCR measurements at 1, 3, or 7 days postcontamination demonstrated that reductions in ASFV nucleic acid level were greater with higher inclusion rates and longer incubation times. All inclusion rates were also effective at reducing viral infectivity and the greatest effect was observed with the highest inclusion rate, which equated to an 88% decrease in viral infectivity. Judging from these results, another interesting observation is that GML (as described above) and formaldehyde had comparable levels of feed mitigation based on infectivity reduction levels.

#### 4.3.4. Other Mitigant Candidates

Another promising mitigant option involves a 1:1:1 blend of natural oils extracted from *Eucalyptus globulus*, *Pinus sylvestris*, and *Lavandula latifolia*, which was found to contain terpenoid-based antiviral molecules such as cineole, linalool, and isobornyl acetate [173]. Initially, different dilutions of the natural oil blend ranging from 250,000 to 8 ppm (starting at 25 v/v% downwards) were tested for PAM cell cytotoxicity, and high viability without cell death was maintained for 72 h at mitigant concentrations up to 1000 ppm [174]. Different oil dilutions were incubated with the virulent ASFV VNUA-ASFV-L01/HN/04/19 strain in aqueous solution before infecting PAM cells with the virus–mitigant mixture and dilutions down to 63 ppm natural oil blend were effective at fully preventing ASFV infection in vitro. The latter result was confirmed by PCR results.which demonstrated markedly lower ASFV nucleic acid levels compared to the virus-only positive control.

While other mitigants have been mainly explored for feed applications, this natural oil blend has been uniquely tested as a drinking water additive at 80 ppm concentration (equivalent to 80 mL/ton) to combat ASFV infection in pigs [141]. The experiments involved the virulent ASFV VNUA-ASFV-L01/HN/04/19 strain and it was found that the drinking water additive prevented lateral transmission from ASFV-infected pigs to cohoused, healthy pigs according to observed clinical symptoms and PCR diagnostics. On the other hand, in the untreated group that did not receive the drinking water additive, ASFV-infected pigs caused cohoused, healthy pigs to also become infected. Among ASFV-infected pigs in the treatment group, the drinking water additive did not prevent ASFV infection but did delay the progression of clinical symptoms.

In addition to natural oils, benzoic acid is a widely used antimicrobial in food applications and has been recently explored as an ASFV mitigant [142]. Various dilutions of pure benzoic acid or combined with a cocktail of natural flavoring agents (thymol, eugenol, piperine, and curcumin) were tested in terms of PAM cell viability and the antiviral properties of a non-toxic dilution were then further evaluated. Benzoic acid alone and in the mixture was effective at inhibiting the virulent ASFV China/GZ20180 strain in aqueous solution based on viral infectivity experiments. The inhibitory effect was further confirmed by reported reductions in ASFV nucleic acid levels. In feed mitigation experiments, both additives were effective at a 0.5% inclusion rate while benzoic acid combined with the natural flavoring agents was the most effective one in terms of speed and magnitude of mitigation. While further research is needed to understand why the inclusion of natural flavoring agents improves ASFV inhibition, similar effects have been reported for pig growth performance gains [175], and it has been discussed how benzoic acid mainly acts as an acidifier whereas the natural flavoring agents contain hydrophobic structural features that may aid membrane disruption [176].

Since ASFV is susceptible to highly acidic environments, a buffered formic acid solution with a pH around 2.6–3.2 has also been explored as a less hazardous alternative to formaldehyde in order to inhibit ASFV contamination of feed ingredients [143]. Different feed ingredients were mixed with a fixed volume of 1 or 2 v/v% formic acid solution and then spiked with the virulent ASFV Georgia 2007/01 strain. There was a dose-dependent effect of the formic acid feed additive on mitigating ASFV nucleic acid levels in maize according to PCR measurements and similar results were also obtained with compound feed and rice bran. However, the mitigant was not effective in all feed ingredients. There was no change in soybean meal and the ASFV nucleic acid levels were unexpectedly higher in meat/bone meal compared to the virus-only positive control. These findings were corroborated by viral titer quantification results obtained using PBMC cultures, and it was discussed how the additive was less effective when treating feed ingredients with higher protein and fat contents, which may be attributed to a greater intrinsic buffering capacity of these ingredients.

There have also been recent efforts to compare the virucidal efficacy of a powdered organic acid blend (OAB)—consisting of formic acid, lactic acid, calcium formate, calcium lactate, and citric acid with pH 2—with bioactive plant extracts from *H. lupulus* flowers (Phyto.A04, liquid form with pH 11) and from *G. glabra* licorice (Phyto.B, powder form with pH 5) to inhibit a virulent ASFV strain (VNUA/HY-ASF1/Vietnam/2019) in spiked feed [144]. PCR test results showed that 0.3% OAB, 1% Phyto.A04, and 0.1–0.5% Phyto. B were all effective at reducing viral nucleic acid levels and dual and triple combination blends of these feed additives had quicker effects. Viral infectivity analysis of the treated feed sample supernatants was performed on PAM cells in vitro and showed that Phyto.A04 and Phyto. B treatments completely inhibited infectious ASFV by day 3 while OAB treatment alone reduced viral infectivity more slowly. Among single treatments, Phyto.A04 was the most effective, while the triple combination treatments caused the largest reduction in viral infectivity after 1-day storage. Overall, the body of evidence from recent mitigation tests supports that physically disruptive mitigants demonstrate high performance based on the combination of observed antiviral efficacy and regulatory acceptable safety profiles worldwide.

To summarize, the three main classes of mitigation strategies covered herein, including thermal treatments, chemically reactive disinfectants, and physically disruptive mitigants, all demonstrate effective ASFV inactivation in different application contexts and can support integrated control and prevention strategies. For example, thermal treatments are typically one-time procedures to inactivate potential ASFV contaminants in feed, feed ingredients, and swill. These treatments can help to prevent transboundary spread of ASFV via feed and feed ingredients into ASF-free regions, while also ensuring safe use of swill and other food wastes in ASF-endemic regions and other areas with strict biosecurity controls to reduce transmission risk. As discussed above, regulatory guidance supports these uses and growing scientific evidence is providing well-defined protocols to facilitate effective decontamination with high confidence. On the other hand, chemically reactive disinfectants offer a more robust approach for routine decontamination of material surfaces around farm settings and can also help to prevent ASFV persistence in soil and animal carcasses. These disinfectants are widely recommended by regulatory agencies, while different disinfectants have particular advantages such as high efficacy, low cost, chemical stability, and safe handling depending on the context. They can be broadly applied in ASF-free and endemic regions to support routine biosecurity practices. Even so, it should be considered that disinfectants often exhibit reduced efficacy in the presence of organic matter and can pose cytotoxicity-related safety concerns that limit their use in direct animal exposure scenarios. Complementing these options, physically disruptive mitigants offer favorable regulatory profiles and can inhibit ASFV in complex biological matrices while also supporting pig health through immune enhancement and prevention of lateral transmission. Unlike thermal treatment and chemical disinfectant strategies, these mitigants can be integrated more readily into feed and drinking water, making them valuable for long-term biosecurity efforts. Hence, there is strong motivation to develop effective mitigation protocols based on these strategies. Towards this goal, recent advances in developing ASFV surrogate models for more widespread testing possibilities are helping to accelerate future innovation and support practical implementation.

## 5. Viral Surrogates for ASFV Mitigation Testing

ASFV has unique biophysical characteristics compared to many commonly studied swine viruses, and these distinguishing features are a major reason why our coverage of different mitigation strategies has focused on studies conducted using authentic ASFV strains. At the same time, there are relatively few scientific laboratories worldwide that are available for ASFV testing due to strict biosecurity requirements, and this gap has fueled research into the development of surrogate virus models to test potential ASFV disinfectants, mitigants, and other countermeasures. For example, the German Veterinary Society (DVG) recommends that modified vaccinia Ankara virus (MVAV) is a suitable representative to serve as an ASFV-mimicking enveloped virus for testing chemical disinfectants.  Table 4 presents an overview of recently tested surrogate viruses that have been used for testing ASFV mitigation strategies and we discuss relevant application examples.

### 5.1. Vaccinia Virus

MVAV is an attenuated strain of vaccinia virus that is used as a vaccine vector [182] and has a double-envelope structure, similar size, and double-stranded DNA genome structure that bears resemblance to ASFV features. Due to these similar characteristics and the accessibility to test MVAV in more conventional lab environments, researchers have explored how chemical disinfectants like peracetic acid and citric acid affect ASFV and MVAV infectivity in contaminated soil samples [183]. Both viruses exhibited comparable stability levels in acidic soil samples, while the disinfectant effect was virus-dependent. For MVAV, 1% citric acid or 0.1% peracetic acid treatment was sufficient for 4 log_10_ (99.99%) inactivation in different soil types. Likewise, 0.1% peracetic acid had strong disinfecting activity against ASFV as well, but 1% citric acid was less effective against ASFV in topsoil samples. These findings support that MVAV is a reasonable model of ASFV for screening purposes but also suggest that ASFV can be more difficult to inactivate in some cases.

MVAV has also proven useful for testing other types of disinfectants such as alkalinizing slaked lime (Ca(OH)_2_), quicklime (CaO) and lime milk (Ca(OH)_2_ in water) and 10% solutions showed high levels of disinfecting activity against both MVAV and ASFV [184]. In addition, a panel of commercial disinfectants was tested against MVAV and ASFV, and it was found that active disinfectants required similar or higher concentrations to effectively inhibit MVAV as compared to ASFV [185]. This finding supports that MVAV can be a suitable ASFV surrogate for disinfectant testing applications, while it should be noted that different methodologies were used for titer quantification of the two viruses.

### 5.2. Pseudorabies Virus (PRV)

Other enveloped viruses like PRV have also been proposed as surrogates for ASFV. Structurally, PRV virus particles have double-stranded DNA genome structures like ASFV as well as similar diameters around 200–250 nm, yet PRV only has a single bilayer envelope that is distinct from the two bilayer envelopes surrounding ASFV. In one study, it was proposed that disinfectant testing against PRV could provide guidance to select disinfectant candidates that might work against ASFV, and four disinfectants were tested consisting of glutaraldehyde decamethylammonium bromide, peracetic acid, sodium dichloroisocyanurate, and povidone-iodine [186]. Consistent with past MVAV testing results, it was reported that peracetic acid had the best performance, and glutaraldehyde decamethylammonium bromide was also noted to work well but required a relatively longer incubation time and its performance was sensitive to temperature. These results support that PRV might be a useful surrogate, but direct comparison testing with ASFV is needed for validating PRV as a surrogate.

### 5.3. *E. huxleyi* Virus

One of the most significant advances in developing ASFV surrogates has been validating the use of the algae-infecting EhV, which also belongs to the NCLDV family that includes ASFV, and both viruses have two bilayer envelopes as well as similar sizes and genome structures. Recent findings have demonstrated that EhV and ASFV have similarly high thermal stabilities, whereas single-envelope PRRSV virus particles are more susceptible to thermal inactivation at relatively lower temperatures [181]. This issue has highlighted the particularly high risk of ASFV persistence compared to other livestock viruses and also led to further investigation of EhV as a surrogate, especially since its infectivity is limited to marine algae and thus EhV has a lower biosecurity risk for laboratory testing. As ASFV is stable in a wide range of feed ingredients, it has been explored whether EhV is also stable in feed ingredients [187]. To simulate practical working environments, EhV was spiked into various feed ingredients and transported across the United States in order to simulate shipping conditions. Over a 23-day transportation period, EhV maintained high stability and was not degraded. A follow-up study investigated EhV stability in feed ingredients over a 120-day storage period and observed high stability—as indicated by only a 0.2 log_10_ reduction in viral nucleic acid content—in the 4–34°C temperature range [188]. It was suggested that long-term storage may be insufficient to reduce the ASFV contamination risk, highlighting the importance of the thermal treatment and mitigation strategies described above. Viewed collectively, progress in the development and validation of viral surrogates for ASFV mitigation testing is still in early days, but promising evidence has already been reported to support the use of particular surrogates for testing different thermal treatments and chemical disinfectants. Further testing of mitigants as well as broader validation in direct comparison studies would reinforce wider use of viral surrogates to expand ASFV-focused testing options, especially at initial stages of the research and development process.

## 6. Conclusions and Outlook

The development of various mitigation strategies has boosted ASFV biosecurity in recent years and helped to fill in capability gaps arising from the lack of widely available vaccines or antiviral drugs. Owing to different mechanisms, each mitigation strategy has particular advantages and the ASFV-specific data covered herein support that high levels of virus inactivation can be achieved by the various mitigation strategies in different application contexts. For example, thermal treatments have proven highly effective at inactivating ASFV in feed matrices although potential recontamination remains a risk. On the other hand, chemically reactive disinfectants are useful for decontaminating contact surfaces, while disinfectants work optimally on pre-cleaned surfaces devoid of organic contaminants like soils or manure and also pose cytotoxicity issues that generally limit animal and human exposure. In the context of virus inactivation in biological matrices, one of the most promising strategies involves the use of physically disruptive mitigants that mainly work by disrupting the lipid envelope surrounding ASFV virus particles. We have provided detailed coverage of these mitigants and there is growing evidence that they can not only inactivate viruses but also have additional functionalities like supporting immune health, minimizing clinical symptoms, and preventing lateral transmission. These capabilities are helping to reshape the view of mitigation strategies from solely virus inactivation to enabling pig health overall and can potentially be used in combination with emerging vaccine and antiviral options as they become available. As we look forward to these future innovation possibilities in the mitigant space, there are also outstanding scientific questions and research needs that must be addressed as follows:

Where do mitigants inactivate viruses? Thermal inactivation strategies based on elevated temperature exposure immediately inactivate viruses in matrices such as feed, while liquid-phase disinfectants similarly inactivate viruses upon surface contact. However, more scientific evidence is needed to understand where mitigants inactivate viruses. This question is especially relevant to feed-delivered mitigants, and it is important to distinguish whether mitigants primarily inactivate viruses in the feed or upon ingestion in pigs. Many mitigants are applied to feed as free-flowing powders and are only active in the hydrated state so it is possible that virus inactivation occurs upon saliva exposure during ingestion. Likewise, other mitigants that are applied in feed as liquids do not disperse completely throughout the feed so they may also work upon ingestion as well. Understanding where mitigants inactivate viruses can lead to improved mitigant compositions and formulations that work more effectively.

Can mitigants inhibit ASFV within infected pigs and improve pig health? Evidence supports that certain mitigants like GML can inhibit other swine viruses such as PEDV in infected piglets and yield positive health outcomes in terms of virus load and anti-inflammatory responses [158]. Among the ASFV-specific data discussed herein, it was further noted that feed-delivered MCFA mitigants can mitigate clinical symptoms of ASFV infection even upon virus exposure [137]. In addition, it was reported that orally delivered natural oil mitigants can prevent lateral transmission from ASFV-infected pigs to cohoused, healthy pigs [141]. These findings and past clinical evidence with other swine viruses call for further testing of selected mitigants on ASFV infections in pig models. In the absence of antiviral drugs, mitigants may prove useful to restrict virus exposure to subclinical levels, support immune health, and prevent lateral transmission.

Can selected mitigants enhance the protective effect of ASFV vaccines? In addition to antiviral properties, certain mitigants such as GML are also known to have immunomodulatory properties. For example, it has been reported that GML can enhance the immune response of piglets that were administered an inactivated PRV vaccine and piglets that received the vaccine and GML were better protected against viral challenge than piglets that received the vaccine alone [189]. Similar effects of vaccine-related immune enhancement have also been reported for protecting against infectious bronchitis virus in broilers [190]. These findings motivate continued exploration of how GML and other mitigants can potentially improve ASFV vaccination responses. As progress continues on the vaccine front, understanding how mitigants and vaccines might work together can improve the development of broader biosecurity strategies aimed at disease prevention for controlling ASFV and other livestock viruses.

Looking forward, these mitigation strategies can be broadly applied to other enveloped swine viruses such as PEDV, PRRSV, classical swine fever virus (CSFV), and swine influenza virus (SIV). Like ASFV, these viruses adversely impact swine health and pork production and their prevention and control rely on similar principles of biosecurity, disinfection, and virus inactivation. As such, lessons learned from ASFV mitigation efforts and ongoing research progress on that front can help strengthen national control programs for other swine viruses as well as support reducing the economic and food security impacts of swine infectious diseases worldwide.

## Figures and Tables

**Table 1:** Application examples of thermal treatments to inactivate ASFV in biological matrices.

Matrix	Conditions	Key results	Refs.
Field crops (wheat, barley, rye, triticale, corn, peas) contaminated with ASFV-infected blood	2 h drying at room temperature or after drying and 1 h exposure to moderate heat at 40–75°C; all tests were performed with ASFV Armenia08 strain	• Drying step at room temperature alone reduced viral infectivity to undetectable levels. • Compared to drying alone, viral DNA was more degraded after thermal treatment at all tested temperatures according to PCR tests. • Optimal viral inactivation in blood-based matrices was achieved at ≥55°C.	[88]

Feed ingredients (meat and bone meal, soybean meal, maize grain)	0–20 min incubation in respective feed ingredient matrix at 60, 70, 80, or 85°C; all tests were performed with ASFV NIAH-BL01-05 strain	• Measured rate of thermal inactivation, which was defined as time required to reduce ASFV infectivity per 1 log_10_ based on first-order kinetics. • Preheated feed ingredients were incubated with ASFV for a certain period, followed by viral titration analysis. • Quicker inactivation occurred at higher temperatures in all matrices and corresponding rates were around 5–7 min, 2–3 min, 1–2 min, and 0.2–1 min at 60, 70, 80, and 85°C, respectively.	[89]

Three swill formula containing different crude fiber, crude fat, moisture, total carbohydrate, ash, and crude protein levels	0–30 min incubation in respective swill formula matrix at 60, 70, 75, or 80°C; all tests were performed with ASFV NIAH-BL01-05 strain	• Preheated swill materials were incubated with ASFV for a certain period, followed by viral titration analysis. • Quicker inactivation occurred at higher temperatures in all matrices and corresponding rates were 23–33 min, 6–11 min, ~2 min, and ~1 min at 60, 70, 75, and 80°C, respectively. • Extrapolated that complete inactivation of ASFV contamination in swill takes up to 119 and 4 min at 70 and 90°C, respectively.	[90]

Porcine serum alone or mixed with straw, wood shavings, feces, or dry feed	Incubation between 1 min and 60 days in respective matrix at different temperatures (5–70°C); all tests were performed with ASFV POL/2015/Podlaskie strain	• Infectious ASFV remained viable in porcine serum for >60 days at temperatures up to 37°C, 2 days at 50°C, 1 day at 60°C, and ≤5 min at 70°C. • Viral DNA was still detectable by PCR assay even when there was no infectious virus remaining. • Presence of organic matter such as feed, straw, or wood shavings accelerated virus degradation, whereas feces had less effect.	[91]

ASFV-spiked liquid porcine plasma	Spray drying of liquid plasma with outlet air temperature set at 80°C, and residence time of ~0.4 s alone or with subsequent ~1 min incubation at 80°C; all tests were performed with ASFV BA71V strain	• Spray drying with 80°C outlet temperature caused an ~2 log_10_ drop in ASFV infectivity within the plasma. • Additional incubation of plasma sample postdrying for 1 min at 80°C caused ASFV infectivity to decrease by over 4 log_10_ in total. • Data supported that ASFV is more heat-stable than other tested viruses but operating conditions are still suitable for effective ASFV inactivation.	[92]

ASFV-spiked liquid plasma	Spray drying of liquid plasma with outlet air temperature set at 71 or 80°C alone or with subsequent 14-day storage period at 4 or 20°C; all tests were performed with ASFV BA71V strain	• Spray drying with 71 or 80°C outlet temperature caused a 2.5–2.8 log_10_ or 3.2–4.2 log_10_ drop in ASFV infectivity within the plasma, respectively. • No infectious ASFV could be detected after 14-day storage period postdrying at 4 or 20°C. • Data support that procedure can inactivate >5 log_10_ ASFV in liquid plasma to undetectable levels, ensuring feed ingredient safety.	[93]

**Table 2:** Evaluation of chemical disinfectants to inhibit ASFV in solution assays.

Disinfectant	Test conditions	Key results	Refs.
Four commercial disinfectants that contain sodium hypochlorite, potassium peroxymonosulfate, glutaraldehyde, or quaternary ammonium compounds as the active ingredient	0.1%–5% products were incubated with ASFV for 30 min; all tests were performed with ASFV BA71V strain	• Products based on glutaraldehyde or quaternary ammonium compounds were highly cytotoxic and precluded from virucidal testing. • Sodium hypochlorite-based product was active at 0.5% and 1% concentrations and caused ASFV infectivity drops of >4 log_10_ but was less effective in the presence of soil-mimicking organic compounds (only 1% worked in that case). • Potassium peroxymonosulfate-based product at 1% concentration was more effective in highly soiled conditions (4 log_10_ drop) but was cytotoxic in standard conditions.	[108]

Formaldehyde, sodium hypochlorite, caustic soda, glutaraldehyde, phenol, benzalkonium chloride, potassium peroxymonosulfate, acetic acid	0.4%–3% products were incubated with ASFV for 30 min; all tests were performed with ASFV BA71V strain	• Optimized protocol was developed to test virucidal properties of cytotoxic disinfectants. • Sodium hypochlorite, glutaraldehyde, caustic soda, and potassium peroxymonosulfate had the best inactivation performance (>4 log_10_ drop). • Benzalkonium chloride, which is a quaternary ammonium compound, glutaraldehyde, and formaldehyde also exhibited virucidal properties but had high cytotoxicity.	[109]

Twenty-four commercial disinfectants, including oxidizing agents, acids, aldehydes, formic acids, and phenols	Various disinfectant concentrations were incubated with ASFV at 4–21°C for 1–30 min; all tests were performed with ASFV BA71V strain	• Disinfectants were tested according to the manufacturer's instructions, including dilution, temperature, and incubation time. • Disinfectants comprising formic acid, phenolic compounds, and oxidizing agents decreased ASFV infectivity by >4 log_10_. • Other disinfectants comprising hydrogen peroxide, aldehyde, and quaternary ammonium compounds also inhibited ASFV to varying extents but had high cytotoxicity.	[110]

Chlorine dioxide	0.2–0.4 μg/mL compound was incubated with ASFV for 1–4 h; all tests were performed with ASFV GD19 strain	• Longer incubation reduced viral infection of PAMs based on fluorescence-based readout. • Virucidal effect also inhibited mRNA expression of viral p72 structural protein. • Higher chlorine dioxide caused greater ASFV inhibition.	[111]

Hydrogen peroxide	3% compound concentration was incubated with ASFV for 5–15 min; ASFV strain was not specified	• PCR testing demonstrated that hydrogen peroxide treatment damaged viral nucleic acids, which were undetectable after 30 min. • ELISA experiments verified ASFV inactivation due to hydrogen peroxide treatment.	[81]

Highly complexed iodine (HPCI) containing polyiodine, and povidone-iodine (PVP-I)	0.25%–5% compound concentrations were incubated with ASFV for 5–30 min in immersion and spray disinfection assays; all tests were performed with ASFV Pig/HLJ/18 strain	• High cell viability (>90%) was maintained at 5% HPCI and 5% PVP-I concentrations. • Relatively high and low ASFV loads were fully inactivated by 5% and 0.25% HPCI, respectively, within 5 min via immersion or spray disinfection. • 5% PVP-I inactivated high ASFV load in 15 min and 0.25% PVP-I was ineffective at fully inactivating a relatively low ASFV load.	[112]

Highly complexed iodine (HPCI) and compound organic acids (COAs), individually and in combination	0.06%–2% compound/mixture concentrations were incubated with ASFV for 5–30 min; all tests were performed with ASFV Pig/HLJ/18 strain or P60-CD2v-EGFP reporter strain	• ASFV inactivation rate of HPCI, COAs, and HPCI and COA mixtures increased at higher concentrations and with longer time durations. • Optimal inactivation performance was achieved with a 5:1 ratio of HPCI and COAs. • Combination index analysis verified that HPCI and COAs synergistically inactivate ASFV.	[113]

Commercial product comprising formaldehyde stabilized with organic acid and surfactant	0.03%–0.2% concentrations were incubated with ASFV for 0–180 min; all tests were performed with ASFV NUA/HYASF1/Vietnam/2019 strain	• 0.2% product caused ≥4 log_10_ reduction in infectious ASFV within 30 min. • 0.03%–0.1% product caused similar inactivation effects within 60 min. • Virus interaction kinetics followed first-order behavior and inactivation speed was quicker at higher product concentrations.	[114]

Iodine and acid mixed solution, potassium peroxymonosulfate, citric acid, sodium dichloroisocyanurate, glutaraldehyde/deciquam, deciquam	0.125%–0.5% compound concentrations were incubated with ASFV for 30–60 min in immersion and spray disinfection assays; all tests were performed with ASFV HLJ/18-DP148R-del reporter strain	• Applied doses of all disinfectants were effective at inactivating ASFV in immersion and spraying formats according to fluorescence-based viral genome expression readout. • Among disinfectants, sodium dichloroisocyanurate was applied at the lowest concentration (0.125%). • Disinfectants were appreciably less active in the presence of organic material contaminants.	[115]

Two commercial products composed of potassium hydrogen peroxymonosulfates, and one product composed of a quaternary ammonium compound	1:200 to 1:800 dilutions were incubated with ASFV at 4 or 20°C for 1–30 min; all tests were performed with ASFV VNUA-ASFV-L01/HN/04/19 strain	• Complete virus inactivation was defined as ≥3 log_10_ reduction. • Potassium hydrogen peroxymonosulfate-based products could fully inactivate ASFV at 1:200 dilution within 5 min after exposure at 4°C but required 30 min at 20°C. • Quaternary ammonium compound-based product could fully inactivate ASFV at 1:400 dilution within 5 min under both temperature conditions, indicating greater potency.	[116]

Cationic surfactant, glutaraldehyde and cationic/anionic surfactants, iodine, glutaraldehyde and cationic surfactant	0.125%–1% compound/mixture concentrations were incubated with ASFV for 5–15 min; all tests were performed with ASFV VNUA-ASFV-L01/HN/04/19 strain	• All disinfectants achieved >4 log_10_ reduction in ASFV infectivity when applied according to the manufacturer's instructions, including dilution and incubation time. • Higher concentrations generally accelerated ASFV infection and corresponding kinetics showed first-order behavior. • Developed quantitative model to compare inactivation efficacy of different disinfectants at similar concentrations, which can be applied to select the contact time for maximal effect.	[117]

Five commercial disinfectants comprising glutaraldehyde or quaternary ammonium compounds as the active ingredient	1:200 to 1:800 dilutions were incubated with ASFV at 4 or 20°C for 1–30 min; all tests were performed with ASFV VNUA-ASFV-L01/HN/04/19 strain	• All disinfectants inhibited PAM viability down to at least 1:6400 dilution, indicating high cytotoxicity. • Some products fully inactivated ASFV (defined as ≥3 log_10_ reduction) at 1:800 dilution within 1 min at both temperatures. • Other products required application at 1:400 dilution for effective ASFV inactivation, while one product was temperature-sensitive.	[118]

Sodium hypochlorite, glutaraldehyde, potassium peroxysulfate, sodium hydroxide, phenol, acetic acid, benzalkonium chloride, formaldehyde	0.4%–3% compound concentrations were incubated with ASFV at −20, −10, or 21°C for 30 min; all tests were performed with ASFV BA71V strain	• Fixed concentrations of each disinfectant were selected based on WOAH recommendations. • Most disinfectants reduced ASFV infectivity by ≥4 log_10_ at all tested temperatures. • Benzalkonium chloride and acetic acid were less effective at sub-zero temperatures.	[119]

Ozonized water prepared using electrolysis	5–20 mg/L ozonized water was incubated with ASFV for 1, 3, 6, or 10 min; all tests were performed with ASFV SY18 strain (wild type) or ΔMGF-EGFP strain (reporter)	• Ozonized water half-life was ~24 h and did not affect cell viability at tested doses. • 5–20 mg/L ozonized water reduced virulent ASFV infectivity by up to 3 log_10_ in a dose-dependent manner. • Virus inactivation was rapid and main effect occurred within 1 min.	[120]

Acidic water (pH 5–6.5) containing chlorine ions, which was treated with electrolysis to generate hypochlorous acid (HOCl) oxidizing agent	Acidic electrolyzed water containing 5–140 ppm free chlorine was incubated with ASFV for 30 min; all tests were performed with ASFV BA71V strain	• Virucidal efficacy depended on soiling condition in water and higher chlorine concentrations were required in more soiled environments. • Electrolyzed water containing 40–60 ppm free chlorine was effective at reducing ASFV infectivity by >4 log_10_ whereas lower concentrations were less inhibitory. • In water containing 5% fetal bovine serum (FBS) to mimic high-level organic soiling conditions, effective ASFV inactivation on the order of >4 log_10_ required at least 80–100 ppm free chlorine.	[121]

Twenty-eight extracts were prepared from plants used in African traditional medicine and from *Rhamnus glandulosa* shrub	12.5–200 µg/mL plant extracts were incubated with ASFV for 60 min; all tests were performed with ASFV Lisbon 60 strain	• Maximum tolerated concentration of each extract was determined by Vero cell cytotoxicity experiments and used for antiviral tests. • Four extracts inhibited ASFV replication in infected cells by >80%. • Six distinct extracts directly inhibited ASFV virus particles by >80%.	[122]

Nine extracts prepared from the leaf, root, and stem portions of the *Ancistrocladus uncinatus* plant	0.0078-1 mg/mL plant extracts were added to ASFV-infected PBMC cells and incubated for 48 h; all tests were performed with ASFV NIG/99 strain	• Antiviral activity depended on the organic solvent used for extraction. • Acetone, dichloromethane and methanol extracts had potent antiviral activity, which was confirmed by PCR experiments. • Study validated antiviral activity of plant extracts while high cytotoxicity and solubility challenges were noted.	[123]

Fourteen oil, hydroglycerin or hydroglycolic plant extracts	0.6%–80% plant extracts were added to ASFV-infected PBMC cells and incubated for 30 min; all tests were performed with ASFV BA71V strain	• Only peppermint extract at 1.05% concentration was effective at reducing ASFV infectivity by >4 log10, including in the presence of organic matter. • Higher peppermint extract concentrations were virucidal but also cytotoxic to the tested Vero cells. • Strawberry and raspberry extracts did not inhibit ASFV yet are known to exhibit antiviral properties against other viruses.	[124]

**Table 3:** Application examples of antiviral mitigants used to inhibit ASFV in feed or in pigs.

Mitigant	Test conditions	Key results	Refs.
GML (C12 MG) or MCFA (C8:C10:C12 individually and in 51:29:7 ratio)	250 µM or 5 mM mitigant concentration in aqueous solution and 0.25–2 wt% inclusion rate in feed; all tests were performed with ASFV BA71V strain	• 5 mM GML, capric acid (C10 FA), or caprylic acid (C8 FA) reduced ASFV infectivity in aqueous solution by greater than 90%. • Total antiviral effect of GML treatment reduced ASFV infectivity in solution by 99.8%. • GML reduced ASFV infectivity in feed by 88% whereas MCFA combination had negligible effect. • GML feed mitigation had no effect on viral nucleic acids by PCR and affected viral p72 surface protein conformation by ELISA.	[134]

GML (C12 MG) + sodium diformate (commercial formulation)	0.3 wt% inclusion rate in feed; all tests were performed with a ASFV strain belonging to p72 genotype II	• Initial feed treatment with GML caused ~81% drop in ASFV infectivity within 1 h. • ASFV infectivity drops in contaminated feed reached 98.8% by day 3 postincubation. • Residual ASFV infectivity was below limit of detection by day 7 postincubation.	[135]

Mixture composed of 10% GML (C12 MG) and 40% C3 MG along with 8% C8, 8% C10, and 12% C12 lactylates	1:2 to 1:200 mitigant dilutions in aqueous solution and 0.25–2 wt% inclusion rate in feed; all tests were performed with ASFV BA71V strain	• 1:10 dilution and more diluted mixtures maintained high cell viability. • 1:50 dilution and less diluted mixtures exhibited virucidal activity against ASFV in solution, with up to >95% infectivity reduction. • Mixture reduced ASFV infectivity in feed by 83% and affected viral p72 surface protein conformation according to ELISA tests.	[136]

37% aqueous formaldehyde or MCFA (C6:C8:C10 in 1:1:1 ratio)	0.03–0.7 wt% mitigant concentration in aqueous solution and 0.33–1 wt% inclusion rate in feed; in vitro tests were performed with ASFV BA71V strain and feed tests were performed with ASFV Georgia 2007 strain	• 0.3% formaldehyde and 0.6% MCFA reduced ASFV infectivity in aqueous solution by >99.9%. • Virus-spiked feed ingredient samples treated with 0.33% formaldehyde or 1% MCFA had no infectious ASFV at 30 days postcontamination. • Viral nucleic acids in feed samples were still detectable by PCR although most samples were non-infective in in vivo pig bioassay.	[137]

MCFA (C8 alone, C6:C8:C10 in a 1:1:1 ratio, and C8:C10:C12 in 1:1:1 ratio)	25–1000 µg/mL mitigant concentration in aqueous solution and 0.125–0.5 wt% in feed; all tests were performed with ASFV Pig/Hanoi/2019/01 strain	• High PAM cell viability was maintained up to 100 µg/mL mitigant concentrations while C6:C8:C10 was more cytotoxic at 200 µg/mL concentration. • All three mitigants showed dose-dependent inhibition of ASFV in feed according to PCR. • Best performance was observed with C6:C8:C10 mixture, with significant drop in ASFV nucleic acid level in feed starting from 0.125% inclusion rate. • No effect on ASFV infectivity in feed was observed for any mitigant treatment according to viral titer measurements.	[138]

Three formaldehyde-based products (commercial formulations)	3 kg/ton inclusion rate in feed; all tests were performed with ASFV VN/Pig/HN/19 strain	• One liquid product caused significant drops in ASFV nucleic acid level starting from day 1 postcontamination while other products had less effect. • The same product caused a drop in ASFV infectivity in feed by day 3 postcontamination and the maximum effect was a 79% decrease. • Another product caused a smaller drop in ASFV infectivity in feed by day 7 postcontamination.	[139]

33% formaldehyde product (commercial formulation)	1–3 kg/ton inclusion rate in feed; all tests were performed with ASFV VN/Pig/Hue/1270 strain	• Product was effective at reducing ASFV nucleic acid levels in feed in a dose- and time-dependent manner according to PCR. • Highest dose was most effective at reducing ASFV infectivity in feed and maximum effect was equivalent to an 88% decrease.	[140]

Blend of *E. globulus*, *P. sylvestris*, and *L. latifolia* oils (1:1:1 ratio)	80 ppm oil was included in drinking water (80 mL/ton); all tests were performed with ASFV VNUA-ASFV-L01/HN/04/19 strain	• All mitigant-fed pigs cohoused with ASFV-infected pigs did not become infected with ASFV according to clinical symptoms and PCR. • Untreated pigs cohoused with ASFV-infected pigs all became infected with ASFV. • Mitigation delayed disease progression by ~2 days in ASFV-infected pigs compared to control group.	[141]

Benzoic acid alone or mixed with thymol, eugenol, piperine, and curcumin (10:0.4 ratio)	0.5 wt% benzoic acid without or with 0.02 wt% mixture inclusion rate in aqueous solution or feed; all tests were performed with ASFV China/GZ20180 strain	• High cell viability was maintained in presence of 10-fold diluted mitigant concentrations in aqueous solution and feed supernatant. • Both mitigant formulations were effective at inhibiting ASFV in aqueous solution. • Benzoic acid with mixture was more effective than pure benzoic acid at inhibiting ASFV in feed according to PCR.	[142]

Liquid formulation containing 61 v/v% formic acid, 18% water, and 20.5 wt/v% sodium formate	1 or 2 v/v% mitigant was applied to feed ingredients; all tests were performed with ASFV Georgia 2007/01 strain	• Mitigant had a dose-dependent effect on reducing ASFV nucleic acid levels in maize according to PCR. • Similar mitigation effects occurred in compound feed and rice brain and there was negligible effect in soybean meal. • Mitigant inclusion increased ASFV levels in meat/bone meal. • Viral infectivity assay confirmed PCR results and differences were attributed to variation in protein content among various feed ingredients.	[143]

Organic acid blend (OAB of formic acid, lactic acid, calcium formate, calcium lactate, citric acid (8.2:1:1.7:11.9:2.5 ratio), liquid-phase extract from *Humulus lupulus* flowers (Phyto.A04), powder extract from *Glycyrrhiza glabra* licorice (Phyto.B)	0.3 wt% OAB, 1 wt% Phyto.A04, and 0.1–0.5 wt% Phyto.B inclusion rate in feed alone or in combination; all tests were performed with ASFV VNUA/HY-ASF1/Vietnam/2019 strain	• All three additives were effective at reducing viral nucleic acid levels in ASFV-spiked feed according to PCR testing. • Infectious ASFV was completely reduced in spiked feed, with Phyto.A04 and Phyto.B exhibiting strong virucidal effects. • Compared to single treatments, combination treatments were quicker acting and tended to cause greater reductions in ASFV levels in spiked feed.	[144]

**Table 4:** Structural comparison of ASFV and other viruses used as surrogates in recent mitigation studies.

Virus	Genome type	Envelope type	Virion diameter
African swine fever virus	Double-stranded DNA	Two bilayer envelopes	200–300 nm (ref. [177])
Vaccinia virus	Double-stranded DNA	Two bilayer envelopes	300–400 nm (ref. [178])
Pseudorabies virus	Double-stranded DNA	Single bilayer envelope	200–250 nm (ref. [179])
*Emiliania huxleyi* virus	Double-stranded DNA	Two bilayer envelopes	180–250 nm (refs. [180, 181])

## Data Availability

Data sharing is not applicable to this article as no new data were created or analyzed in this study.
